# Prospective measurement of outcomes and complications of tibial tuberosity advancement using novel mini plates in small breed dogs

**DOI:** 10.3389/fvets.2023.1268681

**Published:** 2023-10-25

**Authors:** Leah Miller, Karl C. Maritato, Shawn C. Kennedy

**Affiliations:** ^1^MedVet Pittsburgh, Surgery, McMurray, PA, United States; ^2^MedVet Cincinnati, Surgery, Cincinnati, OH, United States; ^3^MedVet Columbus, Surgery, Columbus, OH, United States

**Keywords:** TTA, cruciate, CRCL, small breed, mini TTA, cruciate disease, CrCL rupture

## Abstract

Cranial cruciate ligament (CrCL) disease is a common orthopedic disease in canine patients. Tibial osteotomy procedures for the treatment of cranial cruciate ligament disease in small breed dogs (<15 kg) have previously been limited. A total of 22 client-owned dogs, 26 stifles, with cranial cruciate ligament disease were treated with novel mini-tibial tuberosity advancement plates. The most common intraoperative complications included the need for plate-cage overlap in 7 stifles (26.92%) and screw head fracture in 1 (3.85%). Post-operative complications included tibial tuberosity fracture (3.85%), post-operative medial patella luxation (7.69%), and persistent lameness (7.69%). Of the 26 stifles evaluated in the medium term (>6–12 months) post-operatively, 92.3% had no lameness, with the remaining 7.7% having Grade 1 lameness. A good to excellent clinical outcome was noted in all 26 stifles that underwent TTA with novel mini plates.

## Introduction

Cranial cruciate ligament disease is one of the most common orthopedic disorders in dogs (1, 2). According to Whitehair et al., CrCL rupture is a disease most commonly noted in dogs weighing >22 kg, with Rottweilers, Newfoundlands, and Staffordshire Terriers being the most common breeds represented (3). However, in more recent studies, West Highland White Terriers and Yorkshire Terriers have also been noted to have a predilection in addition to large breed dogs (4). Multiple surgical procedures have been used to address CrCL disease in veterinary patients, including extracapsular lateral suture stabilization repair (ELSS), tibial plateau leveling osteotomy (TPLO), and tibial tuberosity advancement (TTA) (2, 5–11). Extracapsular repair techniques, such as the lateral fabellotibial suture, rely on periarticular fibrosis for long-term joint stability (2). The most common tibial osteotomy procedures, TPLO and TTA, alter the geometry of the joint to eliminate cranial tibial thrust so that functional joint stability is achieved during weight-bearing (5).

The concept of tibial tuberosity advancement was first introduced in 2002 by Montavon and Tepic (7). The goal of TTA is to stabilize the CrCL-deficient stifle at the stance by neutralizing the cranial tibial shear force. The principles behind TTA are rooted in the human biomechanical model, as analyzed and proposed by Nisell (12). In humans, the tibiofemoral compressive force was approximately in the same magnitude and direction as the patellar tendon force. The tibiofemoral shear forces are directed anteriorly or posteriorly, depending on the extension or flexion of the knee joint, respectively.

The “crossover point” refers to the state where tibiofemoral shear forces are effectively neutralized (2). Montavon and Tepic consequently applied this reference to the canine stifle, while assuming that the total joint forces were parallel to the patellar tendon (7). The crossover point in canines is proposed to be angled at 90° to the patellar tendon angle (PTA); with a PTA of <90°, caudally directed tibiofemoral shear forces remain. By advancing the tibial tuberosity cranially to achieve a PTA of ≤90° at full extension, the joint is stabilized (2, 3, 6, 7, 12, 13).

The TTA has been noted to have positive outcomes with regard to improved lameness and return to function. Prior studies on large breed dogs reported a complication rate of 31.5% (12.3% major and 19.3% minor) (6, 7, 10, 13–17), and major complications include tibial fracture, implant failure, patellar luxation, meniscal tears, lick granuloma, and incisional infection/trauma (6). However, with additional experience, more recent studies have noted an overall complication rate of 19%, with major complication rates of 11.4–11.6% and minor complication rates of 7.6% (8, 9). Minor complications include surgical site seroma and patellar tendonitis (6, 8). Additionally, incidental complications observed at follow-up were previously included in the major complication category, such as a non-displaced tibial tuberosity chip fracture, subsequent meniscal tear, and implant failure (6). According to LaFaver et al., owner satisfaction has been documented in the majority of patients, with 97.8% of owners indicating that they would pursue TTA in the opposite leg if it also were affected (6).

Before the start of this study, TTA surgery was limited to dogs over 15 kg, as smaller screw-based implants that properly fit the smaller tibial tuberosities were unavailable. Therefore, at the time of starting this investigation, there were no published studies regarding outcomes in small breed dogs with tibial tuberosity advancements. A potential benefit of the screw-based plate design over the forked design is the ability to contour the plate and alter its orientation during placement. This design allows for more flexibility and appropriate placement than conventional forked implants (9, 18). The purpose of this study was to prospectively evaluate the clinical outcome of dogs weighing up to 15 kg who received TTA surgery with the newly designed, smaller screw-based implants. We anticipated that the small breed dogs would have a similar outcome to their large breed counterparts while undergoing the same surgery.

## Materials and methods

### Animals

The study population consisted of dogs that presented for surgical consultation for CrCL disease. For inclusion in the study, patients had to be small dogs weighing no more than 15 kg and affected by CrCL disease between March 2015 and September 2016 (Table 1). The pathogenesis of CrCL disease in dogs was discussed with the owners.

**Table 1:** Patient demographics.

**Patient**	**Breed**	**Weight (kg)**	**Age (years)**	**Sex**	**Unilateral vs. Bilateral**
1	Bichon Frise	5.6	9	FS	Bilateral staged
2	Cairn Terrier	7.8	7	MN	Bilateral staged
3	West Highland White Terrier	8.4	9	FS	Unilateral
4	West Highland White Terrier	7.2	10	MN	Unilateral
5	Cocker Spaniel	12	7	FS	Bilateral single session
6	Miniature Schnauzer	9.5	9	FS	Unilateral
7	Terrier Mix	9.4	13	FS	Unilateral
8	Scottish Terrier	11.2	9	FS	Unilateral
9	Maltese Mix	6.5	4	MN	Unilateral
10	Havanese	7	4	MN	Unilateral
11	Yorkiepoo	10.9	7	MN	Unilateral
12	Cockapoo	14.9	11	FS	Unilateral
13	Jack Russel Terrier	8.5	1	MN	Unilateral
14	West Highland White Terrier	9.5	12	MN	Unilateral
15	Bichon Frise	11.4	4	MN	Bilateral staged
16	Bichon Frise	7.6	11	FS	Unilateral
17	Coton de Tulear	6.2	8	FS	Unilateral
18	Pomeranian	8.8	8	FS	Unilateral
19	West Highland White Terrier	8.2	9	FS	Unilateral
20	Cavalier King Charles Spaniel	6.8	7	FS	Unilateral
21	Chihuahua mix	7.1	4	MN	Unilateral
22	Shih Tzu	8.2	11	MN	Unilateral
Median		8.3	8.5		

Before obtaining owner consent for the procedure, stabilization options, such as the newly developed implants for tibial tuberosity advancement and extracapsular lateral sutures, were discussed. Signed informed consent forms were obtained from all owners of patients involved in the study. An ethics committee approval was not required at the institution as these patients received a treatment that is standard for their large breed counterparts with similar implants. Dogs with bilateral CrCL tears and concurrent medial patella luxation were not excluded from the study population. Patients who were lost to mid-term follow-up (>6–12 months) were excluded from the study. Details such as signalment, weight, and implants used were recorded. Intraoperative findings and complications and postoperative outcomes and complications were recorded in Table 2.

**Table 2:** Implants, complications, and follow-up.

**Patient**	**Plate used**	**Screw sizes**	**Cage size**	**Meniscal integrity**	**Introp complications**	**Post op complications**	**2 week recheck**	**6 week recheck**	**6 month rehceck**
1	2	1.5/2.4	4.5	Intact	Plate/cage overlap	None	Grade 2 lameness	Grade 1 lameness	No lameness, per owner
2	2	1.5(2.0)/2.4	4.5	Intact	None	None	Grade 2 lameness	Grade 1 lameness	No lameness
3	2	1.5(2.0)/2.0	6	Intact	Plate/cage overlap	None	Grade 2 lameness	Grade 1 lameness	Grade 1 lame
4	2	1.5(2.0)/2.0	4.5	Intact	None	None	Grade 2 lameness	Grade 1 lameness	No lameness
2	2	1.5(2.0)/2.0	4.5	Intact	None	None	Grade 2 lameness	Grade 1 lameness	No lameness
1	2	1.5(2.0)/2.0	4.5	Intact	Plate/cage overlap	None	Grade 2 lameness	No lameness	No lameness, per owner
5	2 and 3	1.5(2.0)/2.0	6	Torn	Screw head fracture	None	Grade 2 lameness	No lameness	No lameness
6	2	1.5(2.0)/2.0	7.5	Intact	None	Tibial tuberosity fracture	Grade 1 lameness	Grade 1 lame (10 weeks)— evidence of fracture healing	No lameness
7	2	1.5(2.0)/2.0	4.5	Intact	None	None	Grade 2 lameness	Grade 1 lameness	No lameness
8	2	1.5/2.0	6	Intact	Plate/cage overlap	None	Grade 1 lameness	Grade 1 lameness	No lameness, per owner
9	1	1.5/2.0	4.5	Intact	None	None	Not performed	Grade 1 lameness	No lameness
10	1	1.5/2.0	6	Intact	Plate/cage overlap	None	Grade 1 lameness	Grade 1 lameness	No lameness
11	2	1.5/2.0	6	Intact	None	None	Grade 2 lameness	Grade 1 lameness	No lameness
12	1	2	6	Torn	None	None	Grade 1 lameness	Grade 1 lameness	No Lameness, complete healing
13	1	1.5/2.0	6	Torn	None	None	Not noted	Grade 1 lameness	No lameness
14	1	1.5/2.0	6	Intact	None	None	Not noted	Grade 1 lameness	No lameness
15	1	1.5/2.0	6	Intact	None	None	Grade 2 lameness	Grade 1 lameness	No lameness on right, cruciate tear on left (new)
15	1	1.5/2.0	6	Intact	None	None	Grade 2 lameness	Grade 1 lameness	No lameness, complete healing
16	1	1.5/2.0	6	Intact	None	None	Grade 2 lameness	Grade 1 lameness	No lameness, complete healing
17	1	1.5/2.0	6	Intact	None	None	Grade 2 lameness	Grade 1 lameness @ 12 weeks	No lameness, complete healing
18	1	1.5/2.0	6	Intact	None	None	Grade 2 lameness	Grade 1 lameness	No lameness, complete healing
19	1	1.5/2.0	6	Torn	None	None	Grade 2 lameness	Grade 1 lameness	No lameness, complete healing
20	1	1.5/2.0	6	Intact	None	None	Grade 1 lameness	Grade 1 lameness	No lameness, complete healing
21	1	1.5/2.0	6	Intact	None	Persistent MPL (Grade 4 to grade 2 at 6 months)	Grade 3 lameness	Grade 1 lameness	Grade 1 lame, grade 2 MPL; Adequate bone healing
22	1	1.5/2.0	6	Intact	Plate/cage overlap	None	Grade 2 lameness	Grade 1 lameness	No lameness, complete healing

Re-evaluations were performed at 2 weeks, 6 weeks, and ≥6 months postoperatively to assess the degree of lameness and incision healing. Subjective radiographic healing, ranging from no healing to bridging of bone formation along three sites, was assessed at 6 weeks and ≥6 months (15). Lameness was assessed using an institutionally established grading scale, ranging from 1 to 5: (1) some weight shifted off the leg when standing but no obvious lameness when moving; (2) weight-bearing lameness when standing and moving; (3) toe touching lameness with minimal weight-bearing when standing and moving; (4) toe touching lameness with minimal weight-bearing when standing and non-weight-bearing lameness when moving; and (5) non-weight-bearing lameness when standing and moving.

Patella luxation was assessed using a grading scale that ranged from 1 to 4: (1) the patella is manually luxated through extension and returns to normal positioning upon release of pressure; (2) the patella is luxated with internal tibial rotation with flexion of the stifle and typically resolves with extension and external rotation of the tibia; (3) the patella is continuously luxated but can be manually reduced; and (4) the patella is permanently luxated and unable to be reduced (2).

### Implants

**Figure 1:**
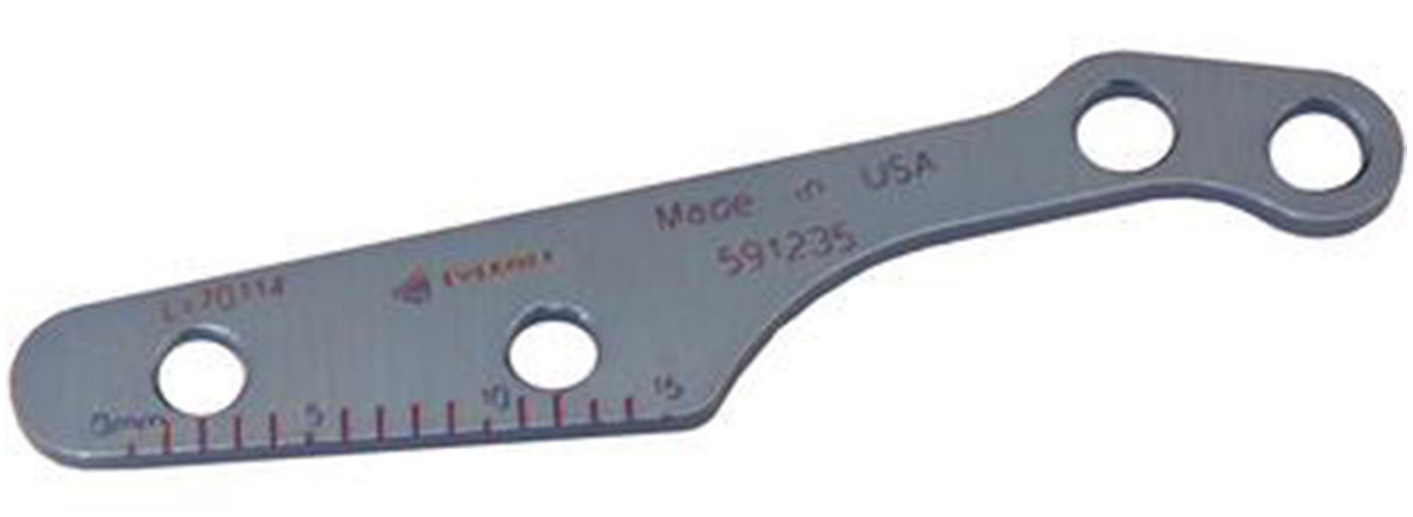
Everost TTA plate.

Screw-based, forkless TTA plates^1^ (Figure 1) were used. Stainless steel 2-hole plates, either #1 or #2, were used. Two 1.5-mm self-tapping screws were used to secure the plate in the tibial tuberosity. Two 2.0-mm self-tapping screws were used in the distal tibia to secure the plate. Stainless steel and bioabsorbable polymer^2^ cages were used, with sizes ranging from 3 mm to 7.5 mm. For patients needing transposition to treat concurrent medial patella luxation (MPL) in addition to the tuberosity advancement, a 6-mm MPL spacer^3^ was used in two patients.

### Surgical procedure

Preoperative blood work (serum biochemistry and complete blood count) was performed within 3 months of the surgical procedure, as regulated by the institution. Patients aged over 8 years were offered thoracic radiographs and a complete abdominal ultrasound prior to surgery as part of geriatric screening. The anesthesiologist on staff determined the anesthetic protocol. Premedications included intravenous hydromorphone^4^ and/or acepromazine.^5^ Induction was performed with various combinations of propofol,^6^ ketamine,^7^ and midazolam,^8^ or alfaxalone.^9^ The patients were intubated and maintained on isoflurane.^10^ A preservative-free morphine^11^ and bupivacaine^12^ lumbosacral epidural were performed in all patients. Preoperative stifle radiographs were made under premedication or general anesthesia based on patient compliance. Radiographs were obtained so as to center on the stifle joint in full extension (6). Measurements for surgical planning were performed so as to determine the distance the tibial tuberosity needed to be advanced to achieve a patellar tendon angle of 90°.

The patient's limb was clipped and aseptically prepped from the proximal thigh to just distal to the tarsus. The patients were placed in dorsal recumbency with the non-surgical leg distracted laterally and the affected limb suspended. The surgery site was draped, ensuring manipulation of the tarsal joint was not impeded. Perioperative antibiotic (Cefazolin 22 mg/kg IV)^13^ was administered a maximum of 30 min prior to the incision and subsequently every 90 min until surgery was completed.

All surgical procedures were performed by a board-certified surgeon. A routine medial approach to the stifle was made; the length of the incision was based on the surgeon's preference. Exploration of the stifle for evaluation of the medial meniscus was performed by either caudomedial arthrotomy or medial parapatellar arthrotomy. In the stifles explored via medial parapatellar arthrotomy, the cruciate ligament was debrided with rongeurs. A meniscal pick was used to assess the integrity of the meniscus. A partial caudal medial meniscectomy was performed on the stifles with caudal medial meniscal tears. In those stifles with an intact meniscus, a meniscal release was performed either by midbody or caudal meniscal tibial release (11). Prior to the closure of the joint, a thorough lavage with sterile saline was performed.

The proximal tibia was exposed, elevating the fascia and pes anserinus for adequate visualization of the proposed osteotomy site. The osteotomy was made parallel to the frontal plane, extending from the distal extent of the tibial crest to the point proximally cranial to the long digital extensor tendon. A 0.4-mm sagittal saw blade was used to make the osteotomy. When possible, the osteotomy was not completed until the plate was secured to the tibial tuberosity. The plate size, either #1 or #2, was determined by proper positioning on the tuberosity so that the cranial aspect of the plate was parallel to the cranial margin of the tibia, with the proximal screw hole aligned with the insertion of the patellar tendon (6). The tuberosity plate screws were placed prior to the completion of the osteotomy, followed by the harvesting of autogenous tibial corticocancellous bone grafts from the tibial metaphysis. The tuberosity was then advanced, with the cage being placed approximately 1–2 mm distal to the proximal edge of the tibia. If a concurrent tibial tuberosity transposition was being performed, a patellar luxation spacer, as previously described, was placed between the cranial wing of the cage and the tibial tuberosity. The tuberosity was transposed such that the patella tracked within the trochlear groove during palpation, manipulation, and range of motion of the stifle and hock with flexion and extension. The osteotomy was held in reduction with an AO-pointed reduction forceps, and appropriate positioning of the tibial tuberosity and cage were confirmed, with mild proximal displacement and distal contact of the tibial tuberosity to the tibia. Before the placement of the screws, cranial tibial thrust was tested and confirmed to be absent. The proximal screws were placed in the wings of the cage, and subsequently, the tibial shaft screws were placed. With the implants secured, cranial tibial thrust was again tested and found to be absent. The surgical site was thoroughly lavaged before the placement of the harvested bone graft in the osteotomy gap. The incision was then closed in a standard fashion. Postoperative radiographs were taken, confirming the appropriate placement of the implants.

All patients were hospitalized overnight for postoperative pain management and were discharged the following day. Bilateral patients were released after 2 days of hospitalization. Postoperative medical therapies included injectable hydromorphone (0.05 mg/kg IV Q 4–6 h)^1^, (Tramadol 2–5 mg/kg PO Q 8 h), various non-steroidal anti-inflammatory medications (unless contraindicated) as needed (Trazodone 3–5 mg/kg PO Q 8–12 h), and Cephalexin 22 mg/kg Q 8 h (19). Additionally, all dogs were discharged with an Elizabethan collar until suture removal, as well as strict exercise restrictions for 6 weeks. Each owner was presented with the opportunity for structured, professional physical rehabilitation.

### Follow up

At 2 weeks postoperatively, each patient's gait and incision healing were subjectively assessed before suture removal. The patients were once again evaluated at 6 weeks for gait analysis, physical examination, and a mediolateral stifle radiograph. As a final evaluation, a physical examination and radiograph of the stifles were performed at 6 months or greater postoperatively.

## Results

A total of 22 dogs (26 stifles) ≤15 kg underwent TTA for stabilization of CrCL-deficient stifles. Four dogs underwent bilateral tibial tuberosity advancements. Three of the four dogs underwent separate bilateral procedures. One patient had a single session bilateral TTA. Two patients with grade 4 MPL underwent concurrent MPL correction. For patients undergoing correction of their MPL, standard techniques of lateral imbrication, medial fascial release, and abrasion sulcoplasty were also performed in addition to tibial tuberosity lateralization with the TTA plate.

Breeds represented included mixed breeds (5); West Highland White Terrier (4); Bichon Frise (3); Cocker Spaniel (2), and one each of the following: Cairn Terrier, Cavalier King Charles Spaniel, Coton de Tulear, Havanese, Jack Russel Terrier, Miniature Schnauzer, Pomeranian, and Shih Tzu. There were 12 spayed females and 12 neutered males. No reproductively intact animals were evaluated. The mean age was 7.77 years (range, 1–13 years), and the mean body weight was 8.75 kg (range, 5.2–14.9 kg) (Table 1).

Surgical complications, both intraoperative and postoperative, were recorded. Complications were categorized as major or minor.

Minor complications were defined as those that do not require subsequent surgical intervention. Major complications are those that require additional surgery. However, no major complications were noted. Of the complications, 50% occurred intraoperatively. Surgical complications can be found in Tables 2, 3. The most commonly noted intraoperative complication was an overlap of the plate and cage, sharing the proximal tibial plate and cranial cage screw holes [7 of 26 stifles (26.92%)]. An overlap of the plate and cage was performed in patients whose tuberosity was too small to fit three screws as in the standardly-performed technique. One of these shared screws had a screw head fracture (3.85%), necessitating repositioning of the plate intraoperatively. Overlap of the plate and cage had no effect on long-term outcomes.

**Table 3:** Complications and outcomes.

**Complications**	**Number of stifles**	**Treatment**	**Outcomes at final recheck**
Plate/cage overlap	7	None	6/7 No lameness 1/7 Grade 1 lameness
Screw head fracture	1	Plate repositioning	No lameness
Tibial tuberosity fracture	1	None, noted at 10-week post-operation radiographs—incidental finding	Evaluated at 15 months post-operation with suspected IVDD and nerve root signature—improved with medical management
Post-operative MPL	2	No additional treatment, improved from Grade 4 to Grade 2	Grade 1 lameness
		No additional treatment	No lameness noted
Mild persistent lameness	2	None	Grade 1 lameness

Persistent grade 1 lameness was noted in two out of 26 stifles (7.69%) at ≥6 months postoperatively. One of these exhibited persistent Grade 2 medial patella luxation (3.85%). Two of 26 stifles (7.69%) were noted to have medial patella luxation at the time of medium-term (>6–12 months) follow-up, one of which was grade 1 lame (Grade 2 MPL), and the other was not lame or reported to have lameness at the time of final follow-up (Grade 1 MPL).

Lameness was evaluated at each recheck that was performed, as noted in Tables 2, 4. At the initial recheck, of 26 stifles, six were noted to be Grade 1 lame, 16 were Grade 2 lame, one was Grade 3 lame, and no patients were Grade 4 or Grade 5 lame. Three stifles were not evaluated at 2 weeks postoperatively. At the second follow-up (6 weeks or equivalent) four had no lameness, 23 had Grade 1 lameness, and one was Grade 2 lame. At the final follow-up (≥6 months), 24 stifles had no lameness, and the remaining two had Grade 1 lameness.

**Table 4:** Patient lameness scores during follow-up.

	**2 weeks post-op**	**6 weeks (or equivalent)**	**≥6 months**
No lameness	0	4	24
Grade 1	6	21	2
Grade 2	16	1	0
Grade 3	1	0	0
Grade 4	0	0	0
Grade 5	0	0	0
Not noted/performed	3	0	0

One stifle (3.85%) showed an incidental healing tuberosity fracture during recheck radiographs at 10 weeks post—operation but exhibited no lameness. A 10-week recheck was performed rather than the standard 6-week recheck due to owner compliance.

## Discussion

Cranial cruciate ligament disease is one of the most common causes of pelvic limb lameness in dogs, with progressive degeneration of the CrCL with subsequent rupture being demonstrated in large breed dogs (20–22). Compared to large breed dogs, dogs weighing <15 kg were noted to have less severe histopathologic changes of the cruciate ligament and decreases in tensile strength of the CrCL, making a degenerative process less likely (22). A gold standard of stifle stabilization for the treatment of CrCL disease has not been established. Historically, in our hospital system, tibial osteotomy-based surgical options for the torn CrCL were not an option for small breed dogs. Rather, they had surgeries such as ELSS. Small breed dogs are commonly noted to have an excessively steep tibial plateau as a result of abnormal caudal angulation of the proximal tibia (23, 24). Therefore, using a tibial osteotomy surgery such as a TTA has benefits over ELSS, especially given that the findings of this study did not find any major complications.

This is a prospective case series involving 22 dogs with 26 stifles weighing up to 15 kg undergoing TTA. The overall complication rate was 50%, with 100% being minor complications. In this study, the plate/cage overlap using one screw in both the proximal tibial tuberosity screw and a dorsal cage was considered a minor intraoperative complication due to previous descriptions of the surgical technique, which calls for the cage and proximal plate screws to be separate. However, our study did not show any clinical impact resulting from the shared use of the screw in our patients. Therefore, if these complications are excluded, the overall complication rate is reduced to 23.07%. This is similar to the overall complication rate in large breed dogs, with a range of 20.0–59% (6, 15, 16). Although the overall complication rate is similar, LaFaver reports a higher major complication rate of 12.3% and a minor complication rate of 19.3% (6).

Minor complications in our study included plate-cage overlap in seven of 26 (26.93%) stifles, screw head fracture in one of 26 (3.85%) stifles, post-operative MPL in two of 26 (7.69%) stifles, and post-operative tibial tuberosity fracture in one of 26 (3.85%) stifles. Previously reported minor complications in large breed dogs included non-displaced tibial tuberosity chip fractures, implant failure, poor graft mineralization, incisional infection/inflammation, and seroma (6, 8). Previously reported major complications include post-liminary meniscal tears, deep surgical site infection, implant failure, patellar luxation, fracture, septic arthritis, excessive screw length, and incisional dehiscence (6, 11, 13, 17). No major complications were noted in our study.

The treatment of concurrent medial patella luxation and cranial cruciate ligament disease via tibial tuberosity transposition advancement has been shown to have good to excellent outcomes (25). One patient in this study that had a persistent MPL and Grade 1 lameness had improved from a Grade 4 MPL to a Grade 2. According to Yeadon, MPL recurrence was noted in 4 of 39 stifles (25). Given the patient's comfort in our study on ranges of motion and the owner's perspective of their quality of life, additional surgery was not performed, and the TTA was considered successful. Though our study has a limited sample size, there is agreement with Yeadon that treatment with TTA with transposition, along with standard treatment, is an option for treating patients with cranial cruciate ligament disease and medial patella luxation (25).

The long-term functional outcome based on follow-up lameness examination, as noted in Table 2, was excellent in 24 of the 26 (92.3%) stifles and good in two (7.7%). This is consistent with previous reports of closing wedge osteotomy (CCWO), TPLO, MMT, and TTA with forked implants in small breed dogs with good to excellent outcomes were noted in 88–100% of patients (10, 17, 26).

Ferreira et al. assessed the use of the previously designed fork-based implants in small breed dogs, excluding patients with medial patella luxation (17). The overall success was similar to that observed in the present study, with 92.3% of the patients having no lameness at the final follow-up. Additionally, they assessed thigh diameter and range of motion (extension and flexion), which were improved to 96, 98, and 97% of the normal limb, respectively. This further supports the clinical success in small breed dogs undergoing TTA. In addition, the current study demonstrates the safety and ability of the screw-based system to concurrently treat MPL.

Limitations of the present study include the absence of objective evaluation of patient outcomes, such as the use of force plate analysis. Moreover, complications were subjectively assessed based on the clinician's opinion that surgical intervention was not warranted. A final limitation was the lack of owner compliance with follow-up examinations and discussions.

Studies by Comeford et al., and Duerr et al. reveal that conservative management of CrCL disease is still widely used in small breed dogs (27, 28). Early studies showed that conservative management could produce acceptable limb function in up to 85% of patients (29, 30). With conservative management, owners reported resolution of lameness within 4 months (range 2.5–5 months) from the time of injury, with 14.3% remaining lame (30). This duration is longer than what is typically seen with surgical intervention. Additionally, Krotscheck et al. (14) showed that osteotomy procedures, such as the TTA and TPLO, have superior objective healing compared to extracapsular repair. Tibial tuberosity advancement and TPLO were shown to achieve normal function at a walk-on gait analysis, while extracapsular repair did not (14). Therefore, we believe that surgical stabilization by an osteotomy should be considered as the primary treatment in these patients rather than ELSS. Previous studies in small breed dogs have specifically reported good quality of life with good to excellent limb function in 94–95% of dogs undergoing the modified Maquet technique (MMT) via TTA Rapid^®^ implants and tibial closing wedge osteotomy for CrCL disease (10, 26). Although ultimate limb function with TTA Rapid^®^ may be similar, the additional complication of tibial fissure/fracture noted with the TTA Rapid^®^ was not observed in our population (10). The decreased number of major complications noted with TTA using plate vs. MMT cage^14^ may suggest a safer option. A potential complication with TTA Rapid and MMP implant removal is a large osteotomy gap requiring additional surgical stabilization. This was noted in practice as a concern by the senior author and is a potential point of study in the future. The positive results of prior studies on small breed dogs further support the use of tibial osteotomy procedures for treatment and improved outcomes for our small breed patients.

The TTA was previously unable to be performed on small breed dogs using the established screw-based implants. The previous screw-based implants posed a challenge due to the size and shape of the tibial crest in comparison to the implants (31). This case series evaluated the use of screw-based implants specifically produced for use on small dogs. This study establishes that TTAs performed in dogs weighing <15 kg can lead to a successful outcome. In conclusion, TTA is an acceptable procedure with good to excellent functional outcomes for stabilization of the CrCL-deficient stifle in dogs weighing up to 15 kg. The complication rate and clinical outcome of dogs ≤15 kg are similar to those of large breed dogs. There were minimal complications, which are common in this type of surgery. Further studies would be necessary to determine if the plate/cage overlap has a clinical effect on patients, though none were appreciated by our population.

## Data availability statement

The original contributions presented in the study are included in the article/supplementary material, further inquiries can be directed to the corresponding author.

## Ethics statement

Ethical approval was not required for the studies involving animals in accordance with the local legislation and institutional requirements because the patients used in this study were receiving a standard treatment for cranial cruciate ligament tears in their large breed counterparts. The patients received the same surgical procedure, pain management and post-operative care. The difference in these patients being the novel mini plates which were created in similar fashion to those used in large breeds. Written informed consent was obtained from the owners for the participation of their animals in this study.

## Author contributions

LM: Data curation, Formal analysis, Writing—original draft, Writing—review and editing. KM: Conceptualization, Methodology, Supervision, Writing—review and editing. SK: Writing—review and editing.

## Footnotes

^1^Forkless TTA plates, Everost Inc., Sturbridge, United States.

^2^Bioabsorpable polymer cage (Xorption), Everost Inc., Sturbridge, United States.

^3^MPL spacer, Everost Inc., Sturbridge, United States.

^4^Hydromorphone 2 mg/ml, West-Ward Pharmaceutical Corp., Eatontown, United States.

^5^Acepromazine 2 mg/ml, SBH Medical LTD, Worthington, United States.

^6^Propofol (Propoflo™) 10 mg/ml, Zoetis, Inc, Kalamazoo, United States.

^7^Ketamine (Zetamine™) 100 mg/ml, VETone^®^, Boise, United States.

^8^Midazolam 5mg/ml, Hospira Inc., Lake Forest, United States.

^9^Alfaxalone (Alfaxan^®^) 10 mg/ml, Jurox Inc., Kansas City, United States.

^10^Isoflurane (Fluriso™) VETone^®^, Boise, United States.

^11^Morphine PF (Astramorph/PF™) 1 mg/ml, Hospira Inc., Lake Forest, United States.

^12^Bupivicaine (Marcaine^®^) 5 mg/ml, Hospira Inc., Lake Forest, United States.

^13^Cefazolin (Ancef^®^) 100 mg/ml, West-Ward Pharmaceutical Corp., Eatontown, United States.

^14^MMT cage (TTA RAPID^®^), Rita Leibinger Medical GmbH & Co. KG, Neuhausen/Tyltlinger, Germany.
